# Psychological distress in patients with restless legs syndrome (Willis-Ekbom disease): a population-based door-to-door survey in rural Ecuador

**DOI:** 10.1186/1756-0500-7-911

**Published:** 2014-12-15

**Authors:** Pablo R Castillo, Robertino M Mera, Paul A Fredrickson, Mauricio Zambrano, Victor J Del Brutto, Oscar H Del Brutto

**Affiliations:** Division of Sleep Medicine, Mayo Clinic, 4500 San Pablo Rd, Jacksonville, FL 32224 USA; Division of Psychiatry, Mayo Clinic, Jacksonville, Florida USA; Gastroenterology Department, University of Vanderbilt, Nashville, Tennessee USA; The Atahualpa Project, Community Center, Atahualpa, Ecuador; School of Medicine, Universidad Espíritu Santo–Ecuador, Guayaquil, Ecuador

**Keywords:** Anxiety, Depression, Ecuador, Epidemiology, Psychological distress, Restless legs syndrome, Stress, Willis-Ekbom disease

## Abstract

**Background:**

Reported prevalence of restless legs syndrome (RLS), also known as *Willis-Ekbom disease* (WED), varies from country to country, and methodologic inconsistencies limit comparison of data. Impact of RLS on quality of life and health has been studied primarily in industrialized countries, particularly Europe and the United States. Many studies have relied exclusively on self-report of symptoms or have assessed only medical populations. Recently, interest has emerged on the impact of WED in rural, underserved populations globally.

**Methods:**

In a population-based survey conducted in rural Ecuador, we assessed the relationship of psychological distress to WED, evaluated with the Depression Anxiety Stress Scales–21. WED was diagnosed through a 2-phase method in which all residents were screened with the International Restless Legs Syndrome Study Group (IRLSSG) questionnaire and all suspected cases were subsequently confirmed through expert medical examination. WED severity was assessed with the IRLSSG rating scale.

**Results:**

Of 665 persons (mean [SD] age, 59.5 [12.6] years; women, 386 [58%]), 76 had depression, 93 had anxiety, and 60 reported stress. Forty persons (6%) had WED, with 15 (38%) having severe disease. In a regression model adjusted for age and sex, the prevalence of depression, anxiety, and stress was about 3 times greater among persons with WED than the general population.

**Conclusions:**

Although cross-sectional data cannot establish causation, this study shows the large behavioral health burden associated with WED in an untreated, rural population.

## Background

Restless legs syndrome (RLS), also known as *Willis-Ekbom disease* (WED), is an underrecognized disorder characterized by an unpleasant urge to move the legs—occurring most often during evening or nighttime—that is partially or completely relieved with movement [1]. Previous studies have shown that patients with WED have higher rates of depression or anxiety disorders than controls [2–5]. The pathogenesis of this association is complex and probably multifactorial, and perhaps it can be explained through the associated sleep disturbances or shared metabolic pathway in WED [6]. Irrespective of its cause, simultaneous occurrence of psychological distress adds substantially to the negative effects of WED [7]. Therefore, prompt recognition and therapy for depression, anxiety, or stress are mandatory to improve the quality of life for patients with WED.

Although publications have reported data on emotional well-being and RLS, only 3 population-based studies have explored the mental health burden in WED through face-to-face evaluations rather than telephonic or Web-based assessments [8–10].

With the exception of 3 Asian studies [3, 6, 11], all currently available studies reporting data on WED and psychological distress were conducted in whites residing in the United States and Europe. Therefore, additional epidemiologic data in persons from other ethnicities and races are needed. Research on RLS and its mental health burden in underserved, native populations has not been conducted. In the present study, we investigated RLS and the prevalence of mood disorders among a native South American population (Amerindians).

The few population-based studies on the prevalence of WED in Latin America have not addressed the issue of associated psychological distress [12–15]. We previously documented that the prevalence of WED in the rural village of Atahualpa is 6% in residents aged 40 years and older [16]. We conducted the present study in the same rural village of Ecuador to assess the prevalence of self-reported psychological distress among persons with WED living in this underserved population. Because of interindividual vulnerability to both WED and psychological distress, our study provides information distinct from the findings of previous European and North American studies, including differences in sleep environment, as well as socioeconomic and demographic characteristics. This information may help to reduce the impact of health disparities through an improved understanding of the contribution of ethnicity and race and sociodemographic characteristics to WED and psychological distress.

## Methods

The Atahualpa Project is a population-based study designed to reduce the burden of neurologic diseases in rural coastal Ecuador [17, 18]. Residents of Atahualpa, Ecuador, are racially homogeneous, with more than 95% belonging to the Mestizo ethnic group, or persons of Native American and mixed European descent, who are also called *Amerindians*. The village is located at sea level, close to the equator (longitude and latitude, 2°18’S and 80°46’W), and was selected for this project because it is representative of the country of Ecuador. The protocol and the informed consent form were approved by the institutional review board of Hospital Clínica Kennedy in Guayaquil, Ecuador (FWA 00006867). Trained field personnel performed a door-to-door survey directed to assess demographic characteristics of all Atahualpa residents aged 40 years and older, defined as persons who had lived in the community for 3 months before June 15, 2013, the day of the survey.

For this first phase of the study, rural physicians interviewed consented persons with a questionnaire directed to identify those with suspected WED. The field instrument used for the detection of suspected cases was developed by the International Restless Legs Syndrome Study Group (IRLSSG) [19]. It consists of 4 questions that must be answered affirmatively to create suspicion of WED. During the second phase, a certified neurologist (O.H.D.B.) and a sleep specialist (P.R.C.) transitorily moved to Atahualpa to medically examine all persons who were screened because of suspected WED, as well as a sample of age- and sex-matched persons considered negative for WED during the screening phase. Whether a person was considered to have a suspected case of WED or to be a control subject was masked to the neurologists. During this phase, persons originally thought to have WED but who instead had a condition that mimicked WED (eg, nocturnal cramps, diabetic neuropathy) were excluded, and those with a definitive diagnosis of WED were inquired about disease severity through use of the IRLSSG rating scale [20]. This scale consists of 10 questions rated on a 4-point Likert scale, with a maximum total score of 40; a score greater than 20 is considered a severe case of WED.

Psychological distress also was evaluated during the survey. For this phase, we used the Depression Anxiety Stress Scales–21 (DASS-21) [21]. This scale provides quantitative measures of depression (dysphoria, hopelessness, devaluation of life, self-deprecation, lack of interest and involvement, anhedonia, and inertia), anxiety (autonomic arousal, skeletal muscle effects, situational anxiety, and subjective experience of anxious affect) and stress (chronic nonspecific psychological arousal, difficulty relaxing, nervous tension, and being easily upset, irritable, and intolerant). The DASS-21 is a reliable and consistent field instrument containing 3 sets of questions (7 questions per set) evaluating the different axes of psychological distress. Each response is rated on a 4-point Likert scale ranging from 0 (not at all) to 3 (almost always) with a maximum total score of 21 for each axis.

One of the 3 field instruments used in our survey, the questionnaire developed by IRLSSG in 2003 for detection of suspected WED, had been previously validated in Ecuadorian Spanish-speaking communities [13]. The other 2 instruments—the IRLSSG rating scale and the DASS-21—were independently translated and back-translated from English to Spanish by bilingual neurologists from our group (O.H.D.B. and P.R.C.). Then, the Spanish versions of both scales were culturally adapted with the aid of Atahualpa’s community leaders and tested in a random sample of the population before the full survey.

Descriptive statistics are presented as mean and SD for continuous variables and as number and percentage for categorical variables. Statistical significance was determined with χ^2^ test for categorical variables and Kruskal-Wallis test for continuous variables. Using a regression model, we examined the association between WED and each axis of psychological distress after adjusting for age and sex. Univariate and multivariate analyses were performed with WED as the dependent variable. All variables were dichotomized, and the output of the model was the odds of a given relation. We also evaluated whether WED was more severe among persons with depression, anxiety, or stress than those who did not test positive for these axes of psychological distress. All analyses were performed using Stata software version 13 (StataCorp LP).

## Results

The door-to-door survey identified 688 Atahualpa residents aged 40 years and older, of whom 23 declined participation or could not complete the questionnaires. In total, 665 persons (mean [SD] age, 59.5 [12.6] years; women, 58%) were interviewed with both the IRLSSG instrument and the DASS-21. The IRLSSG instrument was positive in 94 persons, but neurologic evaluation showed that only 40 had WED. None of the controls were found to have the disease, giving a 6% prevalence of WED (40/665) among Atahualpa residents in this age-group. Mean (SD) age of patients with WED was 53.5 (7.8) years, and 25 (63%) of these participants were women. Patients with WED were younger than the average age of all participants (*P* = .002), but the percentage of women was not significantly different among those with and without the disease (*P* = .65). No participant had previously received a diagnosis of WED, and none had received dopaminergic agonists or any other specific WED therapy.

Results of the DASS-21 indicated that 76 persons had depression, 93 had anxiety, and 60 had stress. None of these persons had received a previous diagnosis of these conditions or had been treated with sedatives or antidepressants. Depressed and anxious persons were older than those without depression or anxiety (*P* = .009 and *P* < .001, respectively), but the prevalence of depression and anxiety was not significantly different across men and women. In contrast, stress was less common in women than in men (*P* = .01), but its prevalence was not influenced by age. No differences were found in the mean age or the sex of persons who had only 1 axis of psychological distress (n = 66) compared with those who had 2 or 3 axes (n = 74). Fifteen (38%) of the 40 patients with WED had a severe disease according to the IRLSSG rating scale. The different axes of psychological distress were not more prevalent among persons with severe WED than those with mild to moderate WED (Table 1).

**Table 1:** **Depression, anxiety, and stress in WED severity in natives and mestizos in rural coastal Ecuador**

		Patients, No. (%)		
Characteristic of functional distress	WED (n = 40)	Severe WED (n = 15)	Mild or moderate WED (n = 25)	***P***value
Depression	12 (30.0)	5 (33.3)	7 (28.0)	.72
Anxiety	12 (30.0)	5 (33.3)	7 (28.0)	.72
Stress	9 (22.5)	3 (20.0)	6 (24.0)	.77
≥2 Axes of functional distress	11 (27.5)	4 (26.7)	7 (28.0)	.93

*Abbreviation*: *WED* Willis-Ekbom disease.

Results from the regression model, showing correlation among the different axes of psychological distress (alone or in combination) and the presence of WED, are detailed in Table 2. Depression, anxiety, and stress, as well as their combination, were significantly more common among persons with WED than the general population, after adjustment for age and sex.

**Table 2:** **Axes of functional distress according to WED in natives and mestizos in rural Ecuador**

	Patients, No. (%)			
Characteristic of functional distress	Participants in series (N = 665)	With WED (n = 40)	Without WED (n = 625)	Statistical significance, OR (95% CI);***P***value ^a^
Depression	76 (11.4)	12 (30.0)	64 (10.2)	4.5 (2.2-9.7); <.001
Anxiety	93 (14.0)	12 (30.0)	81 (13.0)	3.6 (1.7-7.7); .001
Stress	60 (9.0)	9 (22.5)	51 (8.2)	3.3 (1.5-7.6); .004
≥2 Axes of functional distress	66 (9.9)	11 (27.5)	55 (8.8)	4.3 (2.0-9.2); <.001

*Abbreviations*: *OR* Odds ratio, *WED* Willis-Ekbom disease.

^a^Regression model, after adjustment for age and sex.

## Discussion

The findings of this 2-phase study suggest that WED is associated with substantial mood impairment. Our study showed that self-reported symptoms of depression, anxiety, and stress occur in about one-third of persons with WED and are about 3 times more prevalent than in the general population. Despite these data, psychological distress was not more common among persons with severe WED than those with mild to moderate WED. In this regard, our results are similar to those in an Indian study, where depression and anxiety were common among WED patients seen at a sleep clinic, irrespective of WED severity [6]. On this basis, it may be suggested that psychological distress, particularly depression, may be a comorbidity in WED and probably is due to related pathogenetic pathways (ie, dopaminergic deficiency or serotonin alterations) [22]. However, in a door-to-door population-based Turkish study, patients with severe WED were more likely to have higher scores in the Hamilton anxiety and depression scales [3]. Another study, from Germany, also showed a positive correlation between WED severity and psychological distress [2]. Most of the patients in that study were receiving therapy for WED symptoms, and this finding may have been biased for medication adverse effects.

The relation between psychological distress and WED continues to be complex and it is likely bidirectional. Some studies have shown that WED symptoms occur before depression [3, 5] and others have shown the contrary [23]. Yet, by causing sleep loss, the sensory motor discomfort of WED could be the cause of psychological distress [24].

Antidepressants can exacerbate WED symptoms, and medications used for WED (ie, dopaminergic drugs) may have a role in the development of depression and anxiety. Our survey participants had not been treated with either antidepressants or dopaminergic drugs; therefore, our findings were not influenced by medication use. The medication-naïve nature of the population, as well as the population-based design of our study, argues for the strength of our results. However, a potential weakness is the cross-sectional method of the survey, precluding assessment of causality. Further longitudinal studies in these underserved populations are warranted to determine whether 1) psychological distress precedes WED symptoms, 2) WED symptoms influence the occurrence of depression or anxiety, and 3) both conditions are comorbidities sharing similar pathogenetic mechanisms.

## Conclusions

The results of the present study are suggestive that the mental health burden of WED is considerable, and more data on the cause-and-effect relation of this association are needed for planning a rational therapy that improves the psychological burden of affected persons.

## Abbreviations

DASS-21: Depression Anxiety Stress Scale–21
IRLSSG: International Restless Legs Syndrome Study Group
RLS: Restless legs syndrome
WED: Willis-Ekbom disease.
